# Role of Renin-Angiotensin System and Oxidative Stress on Vascular Inflammation in Insulin Resistence Model

**DOI:** 10.1155/2013/420979

**Published:** 2013-01-08

**Authors:** N. F. Renna, C. Lembo, E. Diez, R. M. Miatello

**Affiliations:** ^1^Laboratory of Cardiovascular Research, Area of Pathological Physiology, Department of Pathology, School of Medicine, National University of Cuyo, Centro Universitario 5500, Mendoza, Argentina; ^2^Institute of Experimental Medicine and Biology of Cuyo (IMBECU), CONICET, Mendoza, Argentina

## Abstract

(1) This study aims to demonstrate the causal involvement of renin angiotensin system (RAS) and oxidative stress (OS) on vascular inflammation in an experimental model of metabolic syndrome (MS) achieved by fructose administration to spontaneously hypertensive rats (FFHR) during 12 weeks. (2) Chronic treatment with candesartan (C) (10 mg/kg per day for the last 6 weeks) or 4OH-Tempol (T) (10^−3^ mmol/L in drinking water for the last 6 weeks) reversed the increment in metabolic variables and systolic blood pressure. In addition, chronic C treatment reverted cardiovascular remodeling but not T. (3) Furthermore, chronic treatment with C was able to completely reverse the expression of NF-**κ**B and VCAM-1, but T only reduced the expression. C reduced the expression of proatherogenic cytokines as CINC2, CINC3, VEGF, Leptin, TNF-alpha, and MCP-1 and also significantly reduced MIP-3, beta-NGF, and INF-gamma in vascular tissue in this experimental model. T was not able to substantially modify the expression of these cytokines. (4) The data suggest the involvement of RAS in the expression of inflammatory proteins at different vascular levels, allowing the creation of a microenvironment suitable for the creation, perpetuation, growth, and destabilization of vascular injury.

## 1. Introduction

Inflammation is a ubiquitous pathological process which is central to the development of multiple cardiovascular diseases. Many vascular diseases such as atherosclerosis, restenosis, and transplant vasculopathy are chronic, progressive processes initiated and propagated by local inflammation of large- and medium-sized arteries [1]. This inflammation is mediated by a variety of cell types including macrophages, lymphocytes, endothelial cells (EC), and vascular smooth muscle cells (VSMC). The multiple cell types which participate in vascular inflammation have evolved to produce common cytokines and specific membrane receptors allowing them to transmit their effects into the cell, permitting these diverse cell types to communicate by expression and recognition of multiple pro- and anti-inflammatory cytokines. As such, cytokines and their receptors are the currency of inflammation, and represent attractive targets for therapeutic modalities in numerous vascular inflammatory disorders.

 Synthesis and recognition of cytokines and receptors by both vascular and inflammatory cells allows bidirectional communication between these two systems and demonstrates that, under particular conditions, we can consider vascular cells as an extended participant in the adaptive immune response. Cytokines often act in synergy with other cytokines and frequently share receptor subunits which combine into homodimers or heterodimers with receptors of other cytokines. Cytokines can drive multiple, often simultaneous, cellular processes including mitogenesis, development, gene expression, fibrosis, and chemotaxis [2]. Proinflammatory cytokines most often lead to activation of nuclear factor-(NF-*κ*B) which acts as a “master switch” for transcription of numerous genes, the expression of which may be appropriate, as in host defense, or maladaptive, as in chronic vascular disease [3–5].

The inflammatory nature of atherosclerosis has prompted broad investigation into vascular inflammatory processes, and consequently, proinflammatory signaling mechanisms in the vascular wall have been well characterized [6–9]. Interest has been placed on understanding the potentially protective role of blocking renin-angiotensin system (RAS) and anti-oxidative systems on vascular wall [10]. Such studies that do exist place a strong emphasis on the role of angiotensin and oxidative stress in the metabolic syndrome vascular remodeling pathophysiology.

Spontaneously hypertensive rats (SHR) provide a model of genetic hypertension that allows the study of essential hypertension. The administration of carbohydrate-rich diets to rats can induce insulin resistance, hyperinsulinemia, dyslipidemia, and moderate hypertension. Chronic fructose-fed rats (FFR) provide a useful experimental model for studying the interaction of the factors that shape the metabolic syndrome. This combined model (FFHR) is representative of hypertensive individuals who eat a modern Western diet rich in refined sugars [11]. We postulate that this dual experimental model could be appropriate for extrapolating results to human pathology.

The hypothesis suggests that RAS and oxygen-free radicals are actively involved in the activation of different molecular inflammatory as cytokines, NF-*κ*B, and VCAM-1 generating a microenvironment that allows cardiovascular remodeling.

## 2. Methods

### 2.1. Animals and Experimental Design

All procedures were performed according to institutional guidelines for animal experimentation; protocol was submitted and approved by the Institutional Committee for Laboratory Animal Use and Care (CICUAL) of the School of Medicine-UNCuyo. Thirty-day-old male Wistar Kyoto rats (WKY) and SHR were fed a standard commercial chow diet ad libitum and housed in a room under conditions of controlled temperature (20°C) and humidity, with a 12-hour light/dark cycle during a 12-week experimental period. Candesartan (C) and 4OH-Tempol (T) were administrated to respective groups during the last six weeks.

- Control (W): WKY receiving food and drinking water (DW) ad libitum.
- SHR: receiving food and DW ad libitum.
- Fructose-Fed Rats (FFR): WKY receiving 10% (w/v) fructose (Parafarm, Buenos Aires, Argentina) solution in DW during all 12 weeks.
- Fructose-fed Hypertensive Rats (FFHR): SHR receiving 10% (w/v) fructose solution in DW during all 12 weeks.
- FFHR + C: FFHR receiving 10 mg/kg C by intraesophageal administration.
- FFHR + T: receiving 10^-3 ^M T in DW ad libitum.

At the end of the experimental period, rats were anesthetized with sodium pentobarbital (50 mg/Kg ip), blood samples were taken and arteries and organs were aseptically excised for measurements. 

### 2.2. Systolic Blood Pressure Measurement

Systolic blood pressure (SBP) was monitored indirectly in conscious prewarmed slightly restrained rats by the tail-cuff method and recorded on a Grass Model 7 polygraph (Grass Instruments Co., Quincy, MA, USA). The rats were trained in the apparatus several times before measurement. 

### 2.3. Biochemical Determinations

#### 2.3.1. HOMA Index and Intraperitoneal Glucose Tolerance Test

Fasting plasma insulin was assayed by ACS:180SE automated chemiluminescence system (Bayer, Germany). Plasma glucose levels were assayed using a commercial colorimetric method (Wiener Lab., Argentina). Homeostasis model assessment (HOMA) was used as an index to measure the degree of insulin resistance; it was calculated using the following formula: [insulin (*μ*U/mL) × glucose (mmol/L)/22.5] [12].

Three days before the end of the experimental period, a glucose tolerance test (GTT) was performed. Rats fasted overnight were slightly anesthetized with pentobarbital, and glucose was administered (2 g/Kg ip). Blood samples were taken by tail-bleeding at 0, 30, 60, and 90 minutes after injection to determine plasma glucose concentration. The total area under the curve was calculated as mmol/L/90 min.

#### 2.3.2. Assessment of the Lipid Profile

At the end of the experimental period blood samples were drawn from the animals, after fasting for 12 hours. Total plasma cholesterol, HDL cholesterol and triglycerides were assessed using photocolorimetric enzymatic methods (Wiener Lab., Rosario, Argentina). Data are expressed in mmol/L.

### 2.4. Oxidative Stress Determinations

#### 2.4.1. Measurement of Plasma Thiobarbituric Acid-Reactive Substances (TBARS)

In order to demonstrate the effect of increased oxidative stress at the vascular level, plasma lipid peroxidation was assessed by TBARS concentration. This method was based on the reaction between plasma malondialdehyde, a product of lipid peroxidation, and thiobarbituric acid, as has been previously described [13]. No correction for sample protein content was necessary because of the nature of sample [14].

### 2.5. Measurement of Vascular NAD(P)H-Oxidase Activity

The lucigenin-derived chemiluminescence assay was used to determine NAD(P)H-oxidase activity in a segment of thoracic aorta, as previously described [13]. To assess NAD(P)H-oxidase activity, NADPH (500 *μ*mol/L) was added and chemiluminescence was immediately measured in a liquid scintillation counter (LKB Wallac Model 1219 Rack-Beta Scintillation Counter, Finland) set in the out-of-coincidence mode. Time-adjusted and normalized-to-tissue-weight scintillation counters were used for calculations. Measurements were repeated in the absence and presence of diphenylene iodinium (DPI) (10–6 mol/L), which inhibits flavin-containing enzymes, including NAD(P)H-oxidase [15, 16].

### 2.6. eNOS Activity in Homogenates of Cardiac and Arterial Tissue

The activity of Ca^2+^/calmodulin-dependent endothelial nitric oxide synthase, (eNOS) was measured in mesenteric arteries homogenates and in left ventricle cardiac tissue, by conversion of L-[3H]arginine into L-[3H]citruline. Values were corrected according to protein contents in the homogenates (Bradford method) and to incubation time and are expressed as dpm/mg protein/min. The material obtained from each animal was processed independently [16].

### 2.7. Relative Heart Weight

In order to evaluate cardiac hypertrophy, we measured relative heart weight (RHW). Briefly, heart was separated from the great vessels, dropped into a buffered saline solution (PBS), blotted with tissue paper to remove blood, and weighed. Total heart weight was corrected according to the ratio between heart weight (milligrams) and 100 grams of the total body weight before killing.

### 2.8. Tissue Preservation

Tissue samples for histopathology were processed as has been previously reported [17]. Samples from all rats were used for these observations. Anesthetized animals were briefly perfused with PBS (298 mOsmol/Kg H_2_O, pH 7.40, and 4°C) to clear out the blood. Mesenteric arteries were perfused in vivo with the same solution through the mesenteric artery during 5 min. For histological studies, arteries were also perfused with 4% paraformaldehyde solution for 10 min and fixed by paraffin. Five *μ*m-thick tissue slices were transversely cut across the mesenteric tissue on a microstate (Microm HM, Germany) and processed for histological studies. Similar procedure was applied for heart tissue preservation, by aortic retrograde perfusion. 

### 2.9. Quantitative Histomorphometry to Determine Cardiac Hypertrophy

Histomorphological analyses were conducted on slices from the outer (free) wall of the left ventricle (LV) of the heart. Estimations of cardiomyocyte area were made from sections stained with Masson trichrome solution. Areas with transverse sections of myofibers were selected. The contour of the fibers was then drawn manually. Total myocardiocyte area was expressed as square micrometer (*μ*m^2^).

### 2.10. Arterial Structure

Changes in the structure of arterial walls were assessed by measuring the media layer in mesenteric arteries. Dissected mesenteric vascular beds were fixed in 10% formaldehyde, dehydrated, embedded in paraffin, and later cut in microtome. The slices were dyed and examined as has been previously described [17]. Nontransverse sectioned arteries were excluded from investigation. The lumen to media ratio (i.e., internal diameter to medial thickness) (M/L) was then calculated. Fifty slices from each animal were processed were analyzed to obtain an average value for each rat. Average values were then used for final analysis.

### 2.11. SDS-PAGE and Immunoblot Analysis

Mesenteric tissue was washed in PBS and proteins extracted in cold 20 mM Tris-HCl, pH 7.4, 150 mM NaCl, 10% glycerol, 1% Triton X-100, and a protease inhibitor mixture (P2714, Sigma). After sonication for 15 s (3 times with 10-s intervals) and extraction for 30 min at 4°C, sample extracts were clarified by centrifugation at 14,000 ×g for 20 min and used immediately or stored at −20°C. Proteins were separated on 10% polyacrylamide slab gels and transferred to 0.22-*μ*m nitrocellulose membranes (GE, Germany). Nonspecific reactivity was blocked by incubation for 1 h at room temperature in 5% nonfat dry milk dissolved in washing buffer (PBS, pH 7.6, and 0.2% Tween 20). Blots were incubated with anti-p65 and anti-VCAM-1 antibodies (0.2 *μ*g/mL in blocking solution) for 60 min at room temperature. Horseradish peroxidase-conjugated goat anti-rabbit-IgG and swine anti-goat-IgG dissolved in blocking buffer were used as secondary antibodies (0.25 *μ*g/mL, 45 min at room temperature). Excess first and second antibodies were removed by washing 5 times for 5 min in blocking solution. Detection was accomplished with enhanced chemiluminescence system (ABC, Dako System) and subsequent exposure to Kodak X-AR film (Eastman Kodak) for 5–30 s. 

### 2.12. Immunohistochemistry and Digital Confocal Microscopy (IHC)

*Determination of Transcription Factors (WB)*

Rabbit anti-rat NF-*κ*B p65 subunit [Rel A], C-terminus antibody was obtained from Millipore International Inc. (Amsterdam, Netherlands) (AB1604b), and goat anti-rat VCAM-1 (C-19) antibody was obtained from Santa Cruz Biotechnology Inc. (Santa Cruz USA) (sc-1504). Tissue sections were cut at 3 *μ*m thickness from paraffin-embedded blocks. Deparaffinized sections were used to determine inflammatory response. Tissue was permeabilized in 1% Triton X-100 for 15 min, rinsed well with PBS and blocked with sterile filtered 10% normal rabbit serum for 20 min. All antibody solutions were microfuged for 20 min before use. The antibodies were 1 : 1000 diluted. Primary incubations lasted 1 hour at 21-22°C, followed by extensive washes in PBS with Triton X-100, six times for 5 min each. Secondary antibodies, anti-rabbit IgG TR, and anti-goat IgG FITC (Sigma-Aldrich) were diluted in PBS alone in compliance with the manufacturer's instructions. 

Images were collected with Nikon EZ-C1 3.00 software on a Nikon Diaphot TMD microscope equipped for fluorescence with a xenon lamp and filter wheels (Sutter Instruments, Novato, CA, USA), fluorescent filters (Chroma, Brattleboro, VT, USA), cooled charge-coupled device camera (Cooke, Tonawanda, NY, USA), and stepper motor (Intelligent Imaging Innovations, Inc., Denver, CO, USA). Multifluor images were merged, deconvolved, and renormalized using EZ-C1 3.00 Thumbnailler software.

### 2.13. Measurement of High-Sensitive C Reactive Protein (hs-CRP) Concentration

Plasma HS-CRP concentrations were measured using a turbidimetric assay (Bayer Advia 1650, AG Leverkiusen). Data are expressed in mg/L.

### 2.14. Cytokine Determination by “ChemiArray”

Cytokine expression was assessed by ChemiArray system (rat antibody arrays) (Chemicon International, USA): neutrophil chemotactic cytokine 2 and 3 (CINC-2 and CINC-3), CX3CL1, monocyte chemotactic protein-1 (MCP-1), macrophage inflammatory protein-3 alpha (MIP-3 alpha), nerve growth factor beta (beta-NGF), tissue inhibitor of metalloproteinase-1 (TIMP-1), vascular endothelial growth factor (VEGF), granulocyte-macrophage colony-stimulating factor (GM-CSF), interferon gamma (INF-*γ*), interleukin 1 alpha and beta (IL-1*α*, IL-1*β*), interleukin 4, 6, and 10 (IL-4, IL-6, IL-10) LIX, leptin, and tumor necrosis factor alpha (TNF-*α*). We proceeded, according to the manufacturer's instructions, to block nonspecific reactivity by incubation at room temperature for 1 h with a solution according to the instructive. The membranes were incubated in solutions A and B for 60 min at room temperature. Horseradish peroxidase-conjugated antibodies provided by the manufacturer were used. Excess primary or secondary antibody was removed or after 5 washes of 5 min with washing solution. Detection was performed with chemiluminescence system and subsequent exposure to Kodak X-AR film (Eastman Kodak) for 5–30 s. Citokines were distributed in membranes according the map (Table 1).

### 2.15. Reagents

Unless otherwise noted, reagents were purchased from Sigma Chemical Co., MO, USA.

### 2.16. Statistical and Data Analysis

Data are expressed as mean ± SEM. The statistical significance of data comparison between all groups was assessed by one-way ANOVA followed by Bonferroni post-test. A two-sided *P* value of less than 0.05 was considered significant.

## 3. Results 

### 3.1. Biochemical Determinations

To categorize experimental models we assessed metabolic profile of the different groups. Chronic administration of fructose induced several alterations included in the cluster of risk factors that characterizes MS. The comparison between HOMA index and areas under the GTT curve evidenced that FFR and FFHR developed glucose intolerance, as proven by the significantly increased HOMA index and area values compared to control rats (Table 2).

On the other hand, the animals in FFR and FFHR groups also showed significant differences in the levels of triglycerides and HDL-cholesterol when compared to their controls (Table 2). SHR, FFR, and FFHR groups also showed significant differences in the levels of hs-CRP when compared to WKY. FFHR group showed higher hs-CRP levels than other groups (Table 2). 

Chronic treatment with C significantly reduced the HOMA index and areas under the GTT curve; it also reduced triglyceride levels and HDL-cholesterol, reversing the parameters that comprise the MS. Furthermore T partially but significantly reduced these variables. C significantly reduced the values of hsCRP, while T reduced them only partially. See Table 2.

### 3.2. Systolic Blood Pressure Measurement

Table 2 also shows the time-course of SBP changes along the experimental period. By the sixth week, SBP of FFHR and SHR were significantly increased compared to the control group, and there was also an increase in pressure in the FFR group, lower but still significant. 

C normalized SBP to control values and T partially reduced SBP values. C was more effective and powerful in lowering the SBP. Probably the hypertensive state in this experimental model, although it has a component of endothelial dysfunction, should involve angiotensin 1 receptor (AT1R) in the underlying mechanism. 

### 3.3. Oxidative Stress Determinations

Vascular oxidative status was assessed by measurement of the superoxide producing enzymatic activity and its effects on plasma lipid peroxidation.  Table 3 shows that NAD(P)H-oxidase activity was significantly higher in aortas from FFHR when compared to those from other groups. Plasma TBARS values are shown in Table 3. Plasma TBARS concentration was significantly greater in FFR, SHR, and FFHR than in controls.

In addition, the arterial eNOS activity in the proposed models was analyzed as shown in Table 3: FFHR significantly reduced their enzyme activity, contributing to decrease the production and consequent bioavailability of nitric oxide (NO). 

These results confirm that these experimental models, essentially FFHR, have a significant superoxide production and decreased NO bioavailability. T was effective to reduce superoxide production by reducing the activity of NAD(P)H-oxidase and TBARS, and was also able to normalize eNOS activity (Table 3). Furthermore C, probably by inhibiting the activity of NAD(P)H-oxidase, was also able to achieve these effects, normalizing endothelial oxidative status (Table 3).

### 3.4. Quantitative Histomorphometry for Determining Cardiac and Vascular Hypertrophy

The RHW and myocardiocyte area were significantly higher in FFR, SHR, and FFHR than in control rats, demonstrating myocardial hypertrophy in these experimental models (Table 3). The results of *M/L* ratio calculated in mesenteric arteries are shown in Table 2. FFHR always displayed a significantly reduced *M/L* ratio when compared to the corresponding arteries from WKY; this result was also registered for FFR and SHR groups.

Chronic treatment with T significantly reduced myocardial hypertrophy and vascular remodeling, demonstrating that oxidative stress, by activating different mechanisms, actively participates in the process of cardiovascular remodeling.

However, AT_1_R blockade by C was more effective in reducing these variables. The results demonstrate that intracellular cascade post-AT_1_R activation is fundamental to both cardiac and vascular remodeling processes, not only caused by oxidative stress mechanisms, but also by grown factors.

### 3.5. Determination of Transcription Factors

As shown in Table 4, some inflammatory markers were evaluated in mesenteric arteries, as well as the expression of NF-kappa B and VCAM-1, one of the posttranscriptional products that actively participate in vascular inflammation. Both molecules were determined by IHC. The expression of these molecules in the FFHR group increased significantly compared to control groups. In Table 4, right panel shows a representative image of WB of these antibodies. Average optical density significantly increased in mesenteric artery homogenates from FFHR and FFR groups compared to their controls. It can be observed that the distribution of the *β*-actin marker is similar for all groups.

Moreover, it can be seen in images obtained by IHC in vascular wall of these experimental groups. FFHR shows a large increase in NF-*κ*B expression at the level of EC and VSMC, and also expressed VCAM-1 at subendothelial level.

C completely reduced activation and nuclear translocation of NF-*κ*B (p65 fraction) and VCAM-1 expression. On the other hand, T was not able to significantly reduce the activation of NF-*κ*B and VCAM-1 expression, even though the initial description of the activation of this molecule was by ROS. This could be explained because in FFHR, nuclear factor NF-*κ*B production was more important via superoxide generated by AT1R activation. This finding is very important because blocking AT_1_R at vascular level significantly reduced vascular inflammation. This may also be a determining factor in the reduction of vascular remodeling, as previously shown.

### 3.6. Cytokine Determination by “ChemiArray System”

After evaluation and analysis of vascular remodeling and inflammation and increased hsCRP associated to the FFHR model development, we decided to study the local expression of cytokines (Figure 2). By using the previously described kit, we were able to observe a significant increase in several cytokines in FFHR, including the followings: CINC2, CINC3, VEGF, MIP-3, beta-NGF, VEGF, Leptin, TNF-alpha, INF-gamma, and MCP-1 (Table 3). This finding is the first evidence of the presence of local inflammation at vascular level in an experimental model of insulin resistance such as FFHR. Others cytokines measurable by this kit, especially interleukins, could not be evaluated because the study was focused on the mesenteric tissue homogenate but not on peripheral blood, where most of the interleukins are found. This result shows the significant presence of pro-atherogenic cytokines as CINC2, CINC3, VEGF, Leptin, TNF-alpha, MCP-1, and TIMP-1, other with undetermined significance to vascular level even, as MIP-3 and beta-NGF and other as INF-gamma with antiatherogenic effect.

Chronic treatment with T was ineffective in reversing the expression of cytokines in the mesenteric vascular tree (Table 3).

Chronic treatment with C was able to reverse to control values the expression of proatherogenic cytokines as CINC2, CINC3, VEGF, Leptin, beta-NGF, TIMP-1, and MCP-1 and also significantly reduce MIP-3alpha, TNF-alpha, and INF-gamma expression, demonstrating the important role played by the RAS in vascular inflammation in this experimental model (Table 3).

These results allow us to infer that the activation of cytokines is mediated by a different to redox-sensitive way and AT_1_R receptor was actively involved in expression of cytokines.

## 4. Discussion

**Figure 1:**
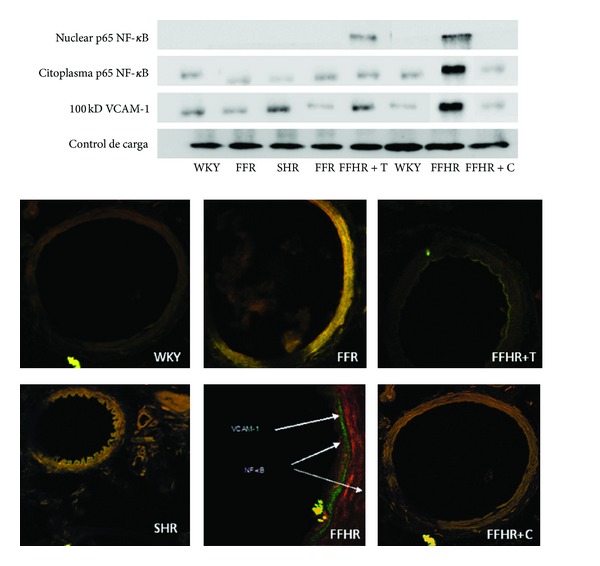
Cytoplasmatic and nuclear p-65 fraction of Nuclear Factor-*κ*B (NF-*κ*B) and Vascular cell adhesion protein 1 (VCAM-1) expression in mesenteric arteries by western blot and Inmunohistochemistry. In up panel shows the western blot representative membrane in which analyzed anti-VCAM-1-FITC and anti-p65-TRITC, the results were obtained by optic density of the bands revealed for each group. In top panel shows microphotographs obtained by laser ICM 600x of mesenteric tissue.

**Figure 2:**
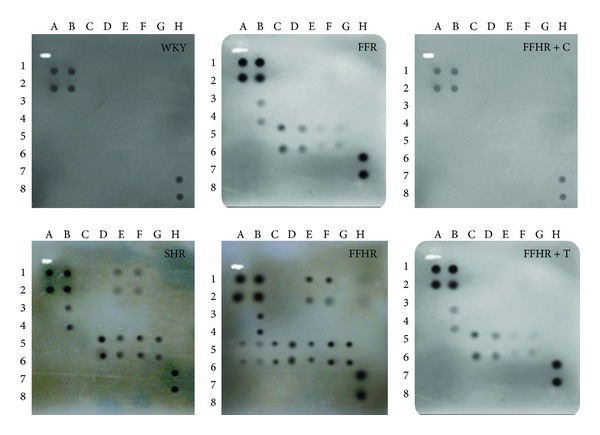
Detection of cytokines on membrane antibody arrays by chemiluminiscence. Each cytokine is represented by duplicate spots in the following locations. See Table 4. Average net light intensity for each pair of cytokine spots detected on the basis of gray-scale levels using US NIH Image software ver. 1.66. Cytokines names: Neutrophil chemotactic cytokine 2 and 3 (CINC-2 and CINC-3), ciliary neurotrophic factor (CNFT), monocyte chemotactic protein-1 (MCP-1), inflammatory protein macrophage-3 alpha (MIP-3 alpha), nerve growth factor beta (beta-NGF), tissue inhibitor of metalloproteinase-1 (TIMP-1) and vascular endothelial growth factor (VEGF), granulocyte colony stimulating factor, macrophage (GM-CSF), interferon gamma (INF-*γ*), interleukin 1 alpha and beta (IL-1*α*, IL-1*β*), interleukin 4, 6, and 10 (IL-4, IL-6, IL-10), lipopolysaccharide induced CXC chemokine (LIX or CXCL5), leptin, and tumor necrosis factor alpha (TNF-*α*).

This study was designed in order to test the hypothesis that suggests the active involvement of RAS and oxidative stress in the activation of different inflammatory molecules as cytokines, NF-*κ*B, and VCAM-1, generating a microenvironment that allows cardiovascular remodeling (Figure 1). We demonstrate the presence of humoral inflammatory markers at vascular level and transcription factors activation, also allows establishing a cause-effect relationship with the AT1R activation.

To our knowledge, this is the first study showing anti-inflammatory effect and inhibition of remodeling progression with a AT1R, candesartan, in an experimental model of MS. AT1-R blocking are associated with reversion of activation of vascular proinflammatory mechanisms found in metabolic syndrome models such as NF-*κ*B expression or cytokine activation. Another important finding is that treatment with a mimetic of superoxide dismutase has similar anti-inflammatory effects to those found with C, although weaker and was not effective in reverting vascular remodeling. Moreover, superoxide blocking production did not overcome protective effects exerted by C.

The experimental model FFHR showed hypertension, dyslipidemia, insulin resistance, vascular and cardiac remodeling, inflammation demonstrated by increased hsCRP and vascular inflammation by increased the NF-*κ*B expression, VCAM-1, and proatherogenic cytokines. The increased expression of VCAM-1, as discussed in the literature, is a marker of vascular inflammation, vascular permeability, and endothelial dysfunction [18].

The inflammatory process found in this experimental model is not only circumscribed at vascular level but it is also systemic, as demonstrated trough the increase in the expression of hsCRP, which is synthesized in the liver in response to increased IL-6. The experimental model showed a very significant increase of this protein [19, 20].

The data suggest the involvement of RAS in the expression of inflammatory proteins at different vascular levels, allowing the creation of a microenvironment suitable for the creation, perpetuation, progression, and unstabilization of vascular injury, be it a simple eutrophic vascular remodeling, or an atherosclerotic lesion.

 A likely explanation is the central role played by the type 1 angiotensin II receptor. Through its intracellular p40 subunit, it is increasing NAD(P)H-oxidase activity, generating an increment of free radicals at both, extracellular and intracellular levels, and activating different cascades of mediators such as PKC, JAK2, PI3 K, FAK, and PLC. These intracellular signal are able to induce translocation of the p65 subunit of NF-*κ*B and AP-1 activation [21] with increased synthesis of cytokines, demonstrated in our work. This activation can also be mediated by oxygen free radicals without depending on the activation of the AT1R but this answer seems to be inferior, with less recruitment of cytokines. Another important point is close relationship with insulin grown factor (IGF) by Src. This signal favoring growth and remodeling of the extracellular matrix, stimulates production of intracellular inflammatory mediators such as NF-*κ*B or AP-1, amplifying the vascular inflammatory response [22, 23].

This study demonstrates the central role of RAS in the pathophysiology of metabolic syndrome. Moreover, it could help to explain some of the findings obtained in large clinical trials.

Our question on these results analysis was whether the superoxide scavenger can restore the bioavailability of nitric oxide or is there another mechanism? This question was answered. Oxidative state is not the unique pathological mechanism involved in the activation of proinflammatory molecules or vascular remodeling, it is also needed an intricate messengers network, able to activate different cascades to enhance and extend endothelial responses to different cells involved in vascular injury process. RAS activates cascades of growth, cell differentiation, and proinflammatory mechanisms, acting as a key in the process of remodeling and vascular injury. 

## Figures and Tables

**Table 1:** Metabolic and cardiovascular variables.

Variable	WKY	FFR	SHR	FFHR	FFHR + C	FFHR + T
Fasting glucose (mmol/L)	4.88 ± 0.1	6.44 ± 0.2*	5.0 ± 0.2	6.5 ± 0.2^∗*∧*^	5.6 ± 0.1**	5.6 ± 0.1**
Fasting triglycerides (mmol/L)	0.8 ± 0.0	1.8 ± 0.0^∗#^	0.9 ± 0.0	1.9 ± 0.1^∗#^	1.1 ± 0.2**	1.7 ± 0.1
HOMA index (*μ*U/mL insulin × mmol/L glucose)/22.5	4.32 ± 0.1	10.93 ± 0.1^∗#^	7.2 ± 0.1*	14.1 ± 0.4^∗#*∧*^	5.7 ± 0.5**	7.2 ± 0.1
Area under glucose tolerance test curve (mmol/L/90 min)	881 ± 64	1392 ± 21^∗#^	1292 ± 31*	1839 ± 51^∗#*∧*^	971 ± 54**	1200 ± 2.4*
HDL-Cholesterol (mg/dL)	22.5 ± 0.7	12.2 ± 0.8^∗#^	19.3 ± 0.9*	13.6 ± 1.2^∗#*∧*^	19.2 ± 1.4**	15.6 ± 2*
High-sensitivity C reactive Protein (mg/dL)	2.55 ± 0.1	3.5 ± 0.0	3.1 ± 0.1	4.5 ± 0.1^∗#*∧*^	2.01 ± 0.0**	4.0 ± 0.0*
Systolic blood pressure (mmHg)						
Baseline	105 ± 3	102 ± 1.0	103 ± 1	105 ± 3	105 ± 2	103 ± 1
6 weeks	113 ± 2.0	131 ± 3.0*	161 ± 3*	162 ± 2^∗#^	165 ± 2*	165 ± 2^∗#^
12 weeks	115 ± 1.3	136 ± 3.0*	177 ± 1^∗#^	181 ± 1^∗#*∧*^	100 ± 2.5**	168 ± 1.1^*∧*∗∗^

The above values correspond to metabolic and cardiovascular variables.

Symbols indicate: *P < 0.001 versus WKY; ^*∧*^ *P* < 0.001 versus SHR; ^#^ *P* < 0.01 versus FFR; **versus FFHR.

**Table 2:** Oxidative stress and morphometric variables.

Variable	W	FFR	SHR	FFHR	FFHR + C	FFHR + T
NAD(P)H oxidase activity (counts/min/mg tissue)	40.5 ± 6	133 ± 5*	160 ± 9.1^∗#^	297 ± 9.1^∗#*∧*^	142 ± 9.1^∗*∧*∗∗^	97 ± 2.1^∗*∧*∗∗^
Arterial eNOS activity (dpm/mg/prot/min)	85.0 ± 2	60.1 ± 2.6*	80.0 ± 2.1	56.4 ± 5.7^∗#*∧*^	86.4 ± 1.1**	84.6 ± 1.1**
TBARS (*μ*mol/L)	1 ± 0.1	2.2 ± 0.1*	1.69 ± 0.1*	2.8 ± 0.1^∗#*∧*^	1.03 ± 0.6^#*∧*∗∗^	0.73 ± 0.4^#*∧*∗∗^
Relative heart weight (mg/100 g body weight)	225 ± 4	290 ± 4*	330 ± 1.8^∗#^	400 ± 4^∗#*∧*^	262 ± 4**	289 ± 4**
Myocardiocyte area (*μ*m^2^)	1682 ± 69	2066 ± 57*	2222 ± 78^∗#^	3242 ± 55^∗#*∧*^	1588 ± 55**	2188 ± 35**
Media/Lumen ratio mesenteric arteries	13.9 ± 0.3	10.2 ± 0.5*	8.9 ± 0.6^∗#^	8.45 ± 0.2^∗#^	14.5 ± 5**	9.5 ± 5**

The above values correspond to stress oxidative and morphometrics variables.

Symbols indicate: *P < 0.001 versus WKY; ^*∧*^ *P* < 0.001 versus SHR; ^#^ *P* < 0.01 versus FFR; **versus FFHR.

**Table 3:** Cytokine release profiles on different experimental models.

Cytokine^a^	Relative levels^b^	Array location^c^	Fold increase for control group (WKY)^d^				
			SHR	FFR	FFHR	FFHR + T	FFHR + C
CINC-2	H	E1-2	1.18	1.10	2.42	2.07	NC
CINC-3	H	F1-2	1.34	1.30	2.74	2.00	NC
CNTF	—	G1-2	~	~	~	~	~
Fractalkine	—	H1-2	~	~	~	~	~
GM-CSF	—	A4-5	~	~	~	~	~
INF-*γ*	H	B3-4	5.00	4.50	5.90	4.00	1.50
IL-1*α*	—	C3-4	~	~	~	~	~
IL-1*β*	—	D3-4	~	~	~	~	~
IL-4	—	E3-4	~	~	~	~	~
IL-6	—	F3-4	~	~	~	~	~
IL-10	—	G3-4	~	~	~	~	~
LIX	—	H3-4	~	~	~	~	~
Leptin	H	A5-6	1.16	1.33	2.10	1.67	NC
MCP-1	H	B5-6	1.25	NC	4.00	2.62	NC
MIP-3*α*	H	C5-6	1.16	1.33	3.91	2.50	1.25
*β*-NGF	H	D5-6	2.40	2.40	3.50	2.93	NC
TIMP-1	H	E5-6	2.60	1.52	2.50	1.90	NC
TNF-*α*	H	F5-6	3.19	1.10	3.35	2.80	1.30
VEGF	H	G5-6	2.50	NC	3.05	2.60	NC

^a^Name of cytokine.

^b^Relative levels: —: undetectable; H: high; L: low.

^c^See Figure 2 for the location of the duplicate spots in the matrix.

^d^When the control “~” symbol was used to indicate an approximation of zero, the values indicate the fold increase versus Wistar Kyoto (WKY: control group). NC: no change (less than or equal to fold difference from the level in WKY group).

**Table 4:** ChemiArray Rat Lysate Cytokine Antibody Array I Map.

	A	B	C	D	E	F	G	H
1	Positive	Positive	Negative	Negative	CINC-2	CINC-3	CNTF	Fractalkine
2	Positive	Positive	Negative	Negative	CINC-2	CINC-3	CNTF	Fractalkine
3	GM-CSF	INF-*γ*	IL-l*α*	IL-l*β*	IL-4	IL-6	IL-10	LIX
4	GM-CSF	INF-*γ*	IL-l*α*	IL-1*β*	IL-4	IL-6	IL-10	LIX
5	Leptin	MCP-1	MIP-3*α*	*β*-NGF	TIMP-1	TNF-*α*	VEGF	Blank
6	Leptin	MCP-1	MIP-3*α*	*β*-NGF	TIMP-1	TNF-*α*	VEGF	Blank
7	Blank	Blank	Blank	Blank	Blank	Blank	Blank	Positive
8	Blank	Blank	Blank	Blank	Blank	Blank	Blank	Positive

Cytokines Names: Neutrophil chemotactic cytokine 2 and 3 (CINC-2 and CINC-3), ciliary neurotrophic factor (CNFT), monocyte chemotactic protein-1 (MCP-1), inflammatory protein macrophage-3 alpha (MIP-3 alpha), nerve growth factor beta (beta-NGF), tissue inhibitor of metalloproteinase-1 (TIMP-1) and vascular endothelial growth factor (VEGF), granulocyte colony stimulating factor, macrophage (GM-CSF), interferon gamma (INF-*γ*), interleukin 1 alpha and beta (IL-1*α*, IL-1*β*), interleukin 4, 6, and 10 (IL-4, IL-6, IL-10), lipopolysaccharide-induced CXC chemokine (LIX or CXCL5), leptin, and tumor necrosis factor alpha (TNF-*α*).
